# Mitochondrial Transfer from Wharton's Jelly Mesenchymal Stem Cell to MERRF Cybrid Reduces Oxidative Stress and Improves Mitochondrial Bioenergetics

**DOI:** 10.1155/2017/5691215

**Published:** 2017-05-04

**Authors:** Yao-Chung Chuang, Chia-Wei Liou, Shang-Der Chen, Pei-Wen Wang, Jiin-Haur Chuang, Mao-Meng Tiao, Te-Yao Hsu, Hung-Yu Lin, Tsu-Kung Lin

**Affiliations:** ^1^Department of Neurology, Kaohsiung Chang Gung Memorial Hospital and Chang Gung University College of Medicine, Kaohsiung 833, Taiwan; ^2^Center of Parkinson's Disease, Kaohsiung Chang Gung Memorial Hospital and Chang Gung University College of Medicine, Kaohsiung 833, Taiwan; ^3^Faculty of Medicine, College of Medicine, Kaohsiung Medical University, Kaohsiung 833, Taiwan; ^4^Center for Translational Research in Biomedical Sciences, Kaohsiung Chang Gung Memorial Hospital, Kaohsiung 833, Taiwan; ^5^Department of Biological Science, National Sun Yat-Sen University, Kaohsiung 833, Taiwan; ^6^Mitochondrial Research Unit, Kaohsiung Chang Gung Memorial Hospital and Chang Gung University College of Medicine, Kaohsiung 833, Taiwan; ^7^Department of Internal Medicine, Kaohsiung Chang Gung Memorial Hospital and Chang Gung University College of Medicine, Kaohsiung 833, Taiwan; ^8^Department of Pediatric Surgery, Kaohsiung Chang Gung Memorial Hospital and Chang Gung University College of Medicine, Kaohsiung 833, Taiwan; ^9^Department of Pediatrics, Kaohsiung Chang Gung Memorial Hospital and Chang Gung University College of Medicine, Kaohsiung 833, Taiwan; ^10^Department of Obstetrics and Gynecology, Kaohsiung Chang Gung Memorial Hospital and Chang Gung University College of Medicine, Kaohsiung 833, Taiwan

## Abstract

Myoclonus epilepsy associated with ragged-red ﬁbers (MERRF) is a maternally inherited mitochondrial disease affecting neuromuscular functions. Mt.8344A>G mutation in mitochondrial DNA (mtDNA) is the most common cause of MERRF syndrome and has been linked to an increase in reactive oxygen species (ROS) level and oxidative stress, as well as impaired mitochondrial bioenergetics. Here, we tested whether WJMSC has therapeutic potential for the treatment of MERRF syndrome through the transfer of mitochondria. The MERRF cybrid cells exhibited a high mt.8344A>G mutation ratio, enhanced ROS level and oxidative damage, impaired mitochondrial bioenergetics, defected mitochondria-dependent viability, exhibited an imbalance of mitochondrial dynamics, and are susceptible to apoptotic stress. Coculture experiments revealed that mitochondria were intercellularly conducted from the WJMSC to the MERRF cybrid. Furthermore, WJMSC transferred mitochondria exclusively to cells with defective mitochondria but not to cells with normal mitochondria. MERRF cybrid following WJMSC coculture (MF+WJ) demonstrated improvement of mt.8344A>G mutation ratio, ROS level, oxidative damage, mitochondrial bioenergetics, mitochondria-dependent viability, balance of mitochondrial dynamics, and resistance against apoptotic stress. WJMSC-derived mitochondrial transfer and its therapeutic eﬀect were noted to be blocked by F-actin depolymerizing agent cytochalasin B. Collectively, the WJMSC ability to rescue cells with defective mitochondrial function through donating healthy mitochondria may lead to new insights into the development of more efficient strategies to treat diseases related to mitochondrial dysfunction.

## 1. Introduction

Mitochondria play crucial roles in oxidative phosphorylation (OXPHOS), ATP production, and diverse cell signaling events. The human mitochondrial genome (mtDNA) is 16,568 bp and encodes a limited number of mitochondria-specific proteins, rRNAs, and tRNAs [1]. Mitochondria are also the major source of intracellular ROS. The electron transport chain (ETC) in the mitochondrial inner membrane is critically involved in the generation of energy, where oxygen acts as an electron acceptor. Mitochondrial ROS is generated in the form of superoxide as a byproduct of inefficient transfer of electrons across the ETC [2]. Accumulated ROS, in a form such as hydrogen peroxide (H_2_O_2_), when built up to a toxic level within cells can result in oxidative stress and oxidative damage [3]. Mitochondria are highly dynamic organelles, able to divide and combine through the processes of fission and fusion, allowing them to adjust their size, shape, and organization inside the cell [4]. Mitochondria dynamic processes are crucial for cell apoptosis, mitochondrial biogenesis, and mtDNA integrity maintenance [5] and are implicated in the aging process [6]. It has been well documented that changes in mitochondrial metabolism affect mitochondrial morphology and network within the cell. Pharmacological inhibition of respiratory chain complexes alters organization of the mitochondrial network, mitochondrial membrane potential, and usually increases ROS production [7, 8]. Increased ROS production triggers dynamic changes in the morphology of the organelles by mitochondrial fragmentation [9]. An intact mitochondrial membrane potential was found to be crucial for mitochondrial dynamics and morphological changes [10]. In mammalian cells, dynamin 1-like (DNM1L or DRP1), mitochondrial fission factor (MFF), and fission 1 (FIS1) are involved in the fission process, while optic atrophy 1 (OPA1) and mitofusin 1 and 2 (MFN1 and MFN2) participate in the fusion process [11].

Mitochondrial DNA (mtDNA) mutations are critical causes of rare human diseases, characterized by mitochondrial dysfunction [12]. Myoclonus epilepsy with ragged-red fiber (MERRF) is associated with specific point mutations of mtDNA, mainly the mt.8344A>G transition in the mitochondrial tRNA^Lys^ coding gene [13], which is associated with severe defects in protein synthesis, leading to impaired OXPHOS [14]. MtDNA mutation-elicited oxidative stress, oxidative damage, and altered gene expression are involved in the pathogenesis and progression of MERRF syndrome [15, 16]. The heteroplasmy ratio of mutated and wild-type mtDNA deteriorates the development and the severity of this syndrome. Clinically, the MERRF syndrome is maternally inherited and is a neurodegenerative disease characterized by myoclonus, mitochondrial myopathy, cerebellar ataxia, and generalized epilepsy. Histologically, Gomori's trichrome staining of MERRF patients' muscle biopsies revealed the presence of ragged-red fibers, due to the subsarcolemmal accumulation of mitochondria [13]. The mt.8344A>G mutation results not only in inefficient bioenergetics but also in intracellular oxidative stress as indicated by an increased expression of ROS and manganese-superoxide dismutase [15]. In addition, cells harboring the mt.8344A>G mutation presented fragmentation of mitochondria, which is correlated with altered processing of the profusion protein optic atrophy type 1 (OPA1, [17]).

More recently, several studies have shown that mitochondrial transfer from stem cells can rescue aerobic respiration of mtDNA-defective cells [18–20] and stressed cells [21–23]. In 2006, Spees et al. first demonstrated that in mtDNA-devoid A549human lung carcinoma cells (*ρ*^0^-A549 cells), cocultured with human bone marrow mesenchymal stem cells (BMMSC), mitochondria moved from BMMSC to *ρ*^0^-A549 cells [18]. Transferred mtDNA from MSC was identified and defective mitochondrial respiration and ATP production of *ρ*^0^-A549 cells were restored [18]. Islam et al. demonstrated that lipopolysaccharide- (LPS-) damaged lung alveoli in vivo received mitochondrial transfer from BMMSC and presented increased ATP restoration, as well as an improved survival rate [21]. In addition, Wang and Gerdes showed that DNA-damaging UV light induced formation of tunneling nanotubes, which facilitate intercellular mitochondrial transfer from healthy cells to damaged cells [23]. Thus, mitochondrial transfer provides a therapeutic opportunity for disorders related to mtDNA defects and mitochondrial dysfunction. Recently, we have shown that human umbilical cord Wharton's jelly mesenchymal stem cells (WJMSC) demonstrate the capability to conduct mitochondrial transfer [23]. *ρ*^0^ cells receiving mitochondria from WJMSC retained the donated mtDNA long term and exhibited restoration of mitochondrial protein synthesis, electron transport chain (ETC) activity, respiratory function, ATP production, mitochondria-dependent growth, and cellular motility [20]. Herein, we report that mitochondrial transfer from WJMSC to MERRF cybrid is able to partly reduce mtDNA mutation load, decrease oxidative stress, and improve mitochondrial bioenergetics. Furthermore, the effect is concomitant with restoration of mitochondrial dynamics and antiapoptosis resistance.

## 2. Materials and Methods

### 2.1. Cell Culture and Cybrid Production

Human 143B osteosarcoma cells were grown in Dulbecco's modified Eagle's medium (DMEM, high glucose, Gibco, Carlsbad, CA, USA) supplemented with 10% heat-inactivated fetal bovine serum (FBS; Gibco, Carlsbad, CA, USA) at 37°C in 5% CO_2_. The FBS was thawed at 37°C and then heated to 56°C for 30 minutes for heat inactivation. Cybrids were generated by fusing 143B-*ρ*^0^ cells with human platelets in the absence of pyruvate and uridine as previously described [24]. Briefly, platelets from healthy volunteers were isolated and fused with *ρ*^0^ cells in the presence of polyethylene glycol 1500 (50% *w*/*v*; Roche, Nutley, NJ, USA). After 1 week of recovery, the cells were placed on a 96-well plate in a 1 : 2 limited dilution in pyruvate/uridine-free medium. After 14 days, the cybrid populations forming single colonies were screened and transferred to a 35 mm dish. With continuing growth, the cybrids were transferred to a 100 mm dish for a further 20 generations of culture in order to ensure that nuclear-encoded mitochondrial proteins were incorporated into the newly introduced mitochondria. Control subject or MERRF patient-donated blood were used for experimentation only after written informed consent had been obtained. This study protocol and written informed content were reviewed and approved by the Institutional Review Board of Chang Gung Memorial Hospital (CGMH; IRB number 101-1620A3).

### 2.2. Isolation, Cultivation, and Identification of WJMSCs

Human WJMSCs were prepared from fresh human umbilical cords obtained during normal spontaneous deliveries after written informed consent had been obtained. The preparation of human WJMSC has previously been described [20]. Briefly, human umbilical cords were placed in Hanks' balanced salt solution (Gibco, Carlsbad, CA, USA) before harvesting of the WJMSC. After the arteries and veins had been removed, the remaining cord was diced into small pieces and transferred to 10 cm dishes containing DMEM in a 37°C incubator at 5% CO_2_. Upon reaching 100% confluence, cells were detached using 0.25% trypsin-EDTA (Gibco, Carlsbad, CA, USA). The WJMSC had a typical spindle-shaped appearance and were found to be positive for CD73, CD90, and CD105 and negative for CD31, CD34, and CD45 in flow cytometric analysis. The WJMSCs used for the experiments were within five passages. Likewise, this study protocol and written informed consent were reviewed and approved by the Institutional Review Board of Chang Gung Memorial Hospital (CGMH; IRB number 101-1620A3).

### 2.3. Mt.8344A>G Mutation Load

To determine the amount of mtDNA containing the mt.8344A>G mutation of MERRF, PCR/RFLP analysis was performed [25]. The primers used were Primer L8150 (8150-8169): 5′-CCGGGGGTATACTACGGTCA-3′ and Primer MR28 (8372-8345): 5′-GGGGCATTTCACTGTAAAGAGGTGCCGG-3′. The amplified PCR products (223 bp) were subjected to restriction digestion by *Nae I* (Thermo Scientific), which can recognize the restriction site (5′-GCCGGC-3′) created by the mt.8344A>G mutation to form a 197 bp and a 26 bp fragment. The PCR products were loaded onto 1.2% agarose gel in Tris-acetate-EDTA (TAE) buffer containing 0.01% of SYBR safe DNA Gen Stain (Invitrogen, Carlsbad, CA, USA). After electrophoresis, the gels were photographed under ultraviolet light. The heteroplasmy of mt.8344A>G was quantified by determining the ratio of 223 bp and 197 bp band intensity using ImageJ.

### 2.4. ROS Expression and Protein Oxidative Damage

Intracellular ROS generation was determined by flow cytometry, following cell staining with a 6-carboxy-2,7-dichlorodihydrofluorescein diacetate (DCFDA) (Sigma) fluorescent probe. DCFDA was converted by oxidation to highly fluorescent 2′,7′-dichlorodihydrofluorescein (DCF), which was detected at an emission wavelength of 530 nm with excitation at 485 nm. Cells were washed twice with PBS and stained with 5 *μ*M DCFDA for 30 min at 37°C. Cell pellets were collected, washed twice with PBS, and then resuspended in PBS. Fluorescence was detected using a FACSCalibur flow cytometer (BD Biosciences, San Jose, CA, USA) and analyzed using CellQuest software (Becton Dickinson, San Jose, CA, USA). For protein oxidative damage, immunoblot-based detection of carbonyl groups, which are introduced into proteins by oxidative reactions with ozone or oxides of nitrogen or by metal-catalyzed oxidation, were detected using Protein Oxidation Detection Kit (OxyBlot™, Millipore).

### 2.5. Western Blot

Cells were lysed in RIPA lysis buffer (50 mM Tris, pH 7.4; 150 mM NaCl; 1 mM phenylmethanesulfonyl fluoride; 1 mM ethylenediaminetetraacetic acid (EDTA); 1% Triton X-100; 1% sodium deoxycholate; and 0.1% SDS) with the addition of Protease Inhibitor Cocktail (Roche Diagnostics, Penzberg, Germany) and Phosphatase Inhibitor Cocktail I (Sigma, St. Louis, MO, USA). The antibodies used included anti-COX2 (Abcam, Cambridge, UK), anti-*β*-actin (Cell Signaling, Danvers, MA), anti-OPA1 (Santa Cruz Biotechnology), anti-MFN2 (Santa Cruz Biotechnology), anti-DRP1 (Santa Cruz Biotechnology), anti-FIS1 (Santa Cruz Biotechnology), and peroxidase-labeled anti-rabbit IgG (H + L) secondary antibody (Abcam, Cambridge, UK). The signals were developed using ECL plus (GE Healthcare Bio-Sciences AB, Uppsala, Sweden) using X-ray films.

### 2.6. Mitochondrial Membrane Potential

Cationic fluorescent dye tetramethylrhodamine ethyl ester (TMRE; Sigma, St. Louis, MO, USA) was used to assess mitochondrial membrane potential as previously described [24]. Cells were incubated in 100 nM TMRE for 30 min at 37°C. They were then washed twice with 1x PBS and fluorescence signal was examined and photographed by fluorescence microscope. Fluorescent intensity of the image was exhibited by HeatMap Histogram, a plugin for ImageJ. For quantitative measurement, cells stained by TMRE or JC-1 (T-3168; Thermo Fisher Scientific) were analyzed using flow cytometry.

### 2.7. Oxygen Consumption Rate (OCR)

OCR was monitored with a Clark electrode (Mitocell S200 micro respirometry system; Strathkelvin Instruments, Motherwell, UK) as previously described [20]. Cells (100 *μ*l at 5 × 10^6^ cells/ml) in KCl medium (100 mM KCl, 3 mM MgCl_2_, 20 mM HEPES, 1 mM EDTA, 5 mM KH_2_PO_4_, pH 7.4) were permeabilized by digitonin (optimal concentration of 32.5 *μ*g/ml determined by trypan blue staining) and loaded into a 200 *μ*l MT200 Respirometer Chamber, suspended by a fixed speed solid-state magnetic stirrer inside the chamber, and maintained at 37°C using a circulating water bath. Basal, ADP-activated, ATP synthase-inhibited, and maximal OCR were determined by adding 10 mM glutamate and 10 mM malate, 0.2 mM ADP, 1 *μ*M oligomycin, 1 *μ*M carbonyl cyanide-4-(trifluoromethoxy)phenylhydrazone (FCCP), and rotenone.

### 2.8. ATP Assay

7.5 × 10^4^ cells were trypsinized, washed, and resuspended in DPBS (Invitrogen, Carlsbad, CA, USA); supplemented with 2% FBS; and incubated in the presence of DMSO or oligomycin (Sigma, St. Louis, MO, USA) at37°C for 2 h. Cells were then collected to determine ATP level (ATP Assay Kit #K354-100, BioVision, Palo Alto, CA, USA) according to the manufacturers' instructions.

### 2.9. Mitochondria-Dependent Viability Assay

2 × 10^4^ cells were seeded in a 24-well plate with glucose-free DMEM supplemented with 10 mM galactose and 10% FBS at day 0. Bright field cellular morphology was observed under a microscope (Leica, Wetzlar, Germany) at days 1 and 2. Viable cell number was counted by trypan blue assay. For general proliferative rate, cells were seeded in high-glucose DMEM (Gibco, Carlsbad, CA, USA) and trypan blue assay was wielded at days 1 and 2.

### 2.10. Experimental Procedures for Coculturing and Imaging

Prior to coculture, mitochondria of WJMSC and cybrid were traced by 500 nM Mitotracker Red FM (Invitrogen, Carlsbad, CA, USA) and 100 nM Mitotracker Green FM (Invitrogen, Carlsbad, CA, USA), respectively, for 45 min at 37°C. After washing three times with PBS, the cells were trypsinized and cocultured on glass coverslips for 24 h. The cocultured cells were then fixed with absolute methanol for 10 min on ice, followed by nuclear staining using 1 *μ*g/ml Hoechst 33342 (Invitrogen, Carlsbad, CA, USA) for 5 min at room temperature, and finally mounting on glass slides for analysis using a fluorescence microscope (Leica, Wetzlar, Germany). In an additional experiment using mitochondria-targeted fluorescent protein, Cox4-DsRed and Su9-EGFP constructed plasmids were, respectively, transfected into WJMSC and MERRF cybrids using Lipofectamine3000 Transfection Reagent (Invitrogen, Carlsbad, CA, USA).

For contact coculture, MERRF cybrid and WJMSC (5 × 10^3^ cells each) were mixed and grown onto a glass coverslip in a 35 mm dish. For separated coculture, WJMSC (upper well) and cybrid (lower well) were separated by 3 *μ*m pore transwell insert system (SPL Lifescience, Pocheon, Korea). After 24 h, the upper insert was removed and cybrid in the lower well was analyzed for image, genotyping, and functions.

### 2.11. Quantification of Mitochondrial Transfer

The interaction of Cox4-DsRed-expressing WJMSCs and Su9-EGFP-expressing *ρ*^0^/cybrid cells were facilitated by direct cocultured. After 24 h, mixed cell population was assayed by flow cytometry. Percentage of mitochondrial transfer was determined by calculating events using the equation below: upper  right/(upper right + upper  left + lower  right) × 100%.

### 2.12. Mitochondrial Morphology

To observe mitochondrial morphology, cells expressing Cox4-DsRed were seeded onto a 35 mm glass bottom dish. Images were taken under a confocal microscope (FluoView FV10i, Olympus). A cell containing only spherical mitochondria was defined as fragmented. A cell with mitochondrial length less than 5 *μ*m was defined as short tubular. A cell with mitochondria more than 5 *μ*m was indicated as long tubular. 50–100 cells were counted for each cell type. The basis for analyzing mitochondrial aspect ratio, elongation, and interconnectivity using ImageJ macro was previously described by Dagda et al. [26]. Aspect ratio calculated by major/minor axis ratio was used to determine the roundness of mitochondria. Mitochondrial elongation was measured by inverse of circularity. Mitochondrial interconnectivity was estimated by area/perimeter ratio.

### 2.13. Determination of Cell Apoptosis

Annexin V fluorescent staining was utilized to determine cell apoptosis. Cells were cultured in medium in the presence of 500 nM staurosporine (Sigma, St. Louis, MO, USA). After 6 h of treatment, cells were washed twice with 0.01 M PBS and once with 1x Annexin V Binding Buffer. Cells were then incubated with Annexin V-FITC diluted 1 : 10 in 1x Annexin V Binding Buffer for 15 min at RT. Annexin V-FITC fluorescence was immediately observed under a fluorescence microscope (Leica, Wetzlar, Germany).

For quantitative determination of apoptosis, cells were harvested with trypsinization at the 90% confluent after indicated treatment, stained by Annexin V-FITC, and then assessed for cell apoptosis by flow cytometry (Beckman Coulter, CA, USA).

### 2.14. Statistical Analysis

Data collected from at least three independent experiments are expressed as the mean ± SEM. Differences between two data sets were evaluated by two tailed unpaired Student's *t*-test. Statistical tests between multiple data sets were analyzed using a one-way analysis of variance (ANOVA) followed by post hoc Bonferroni's test. A *p* value < 0.05 was considered statistically significant.

## 3. Results

### 3.1. MERRF Cybrid Presents High Level of mtDNA Mutation Load, ROS Expression and Oxidative Damage, and Reduced Mitochondrial Bioenergetics

**Figure 1:**
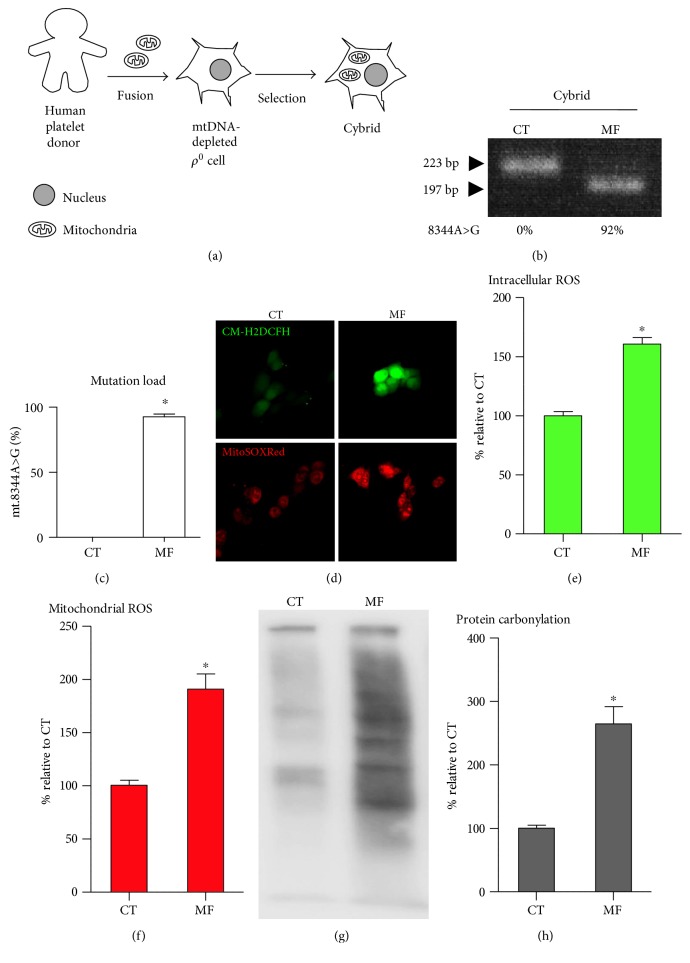
MERRF cybrid with high rate of mt.8344A>G presented higher level of ROS expression and oxidative damage. (a) Schematic drawing shows that cybrid is generated by introducing mitochondria-containing human platelet into mtDNA-depleted *ρ*^0^ cell. (b-c) Mt.8344A>G heteroplasmic rate was examined using PCR-RFLP. PCR fragment containing normal mt.8344A was not digested by *Nae I* and showed a 223 bp band. Mt.8344A>G mutation was *Nae I*-cleaved into 197 bp and 26 bp (not shown in gel). The proportion (%) of mt.8344A>G was quantified using ImageJ. Quantified histogram of mt.8344A>G was obtained by three independent clones. (d–f) Intracellular and mitochondrial ROS expression probed, respectively, by CM-H2DCFH and MitoSOXRed were imaged by fluorescent microscope and quantified by flow cytometry. (g) Protein carbonylation was determined using OxyBlot method. ^∗^*p* < 0.05.

**Figure 2:**
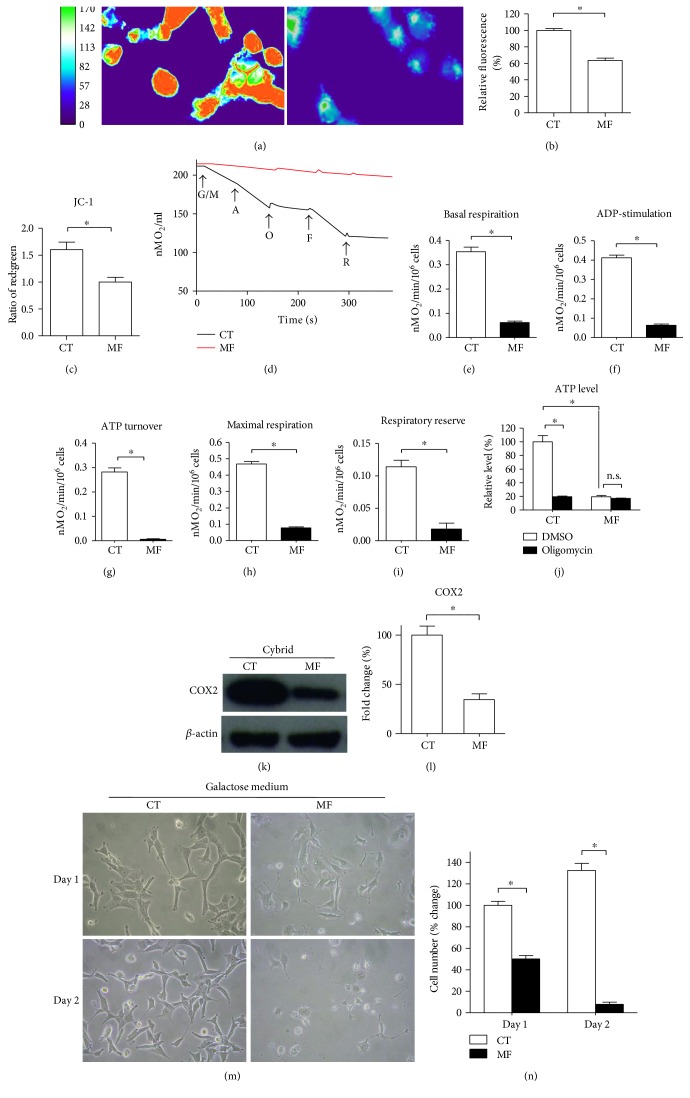
MERRF cybrid demonstrated defective mitochondrial bioenergetics. (a) Mitochondrial membrane potential probed with TMRE was imaged using a fluorescence microscope. Heat map reflecting the fluorescent intensity was analyzed using ImageJ. (b) Quantitative TMRE fluorescence was analyzed using flow cytometer. (c) Mitochondrial membrane potential probed with JC-1 was analyzed using flow cytometer. (d) Representative polarography of OCR measurement. Substrates and inhibitors were sequentially added to cell-containing oximeter chamber, including glutamate/malate (G/M), ADP (A), oligomycin (O), FCCP (F), and rotenone (R). (e) Basal respiration was calculated by OCR in the presence of G/M. (f) ADP-stimulated respiration was calculated by OCR in the presence of ADP. (g) Respiration related to ATP turnover was determined by the difference between ADP-stimulated OCR and oligomycin-suppressed OCR. (h) Maximal respiration was calculated by OCR in the presence of FCCP. (i) Respiratory reserve was determined by the difference between maximal and basal OCR. (j) ATP level was measured in the presence of DMSO or oligomycin. (k-l) Cytochrome c oxidase subunit 2 (COX2) expression level was determined using immunoblotting. *β*-Actin as a loading control. (m-n) Mitochondria-dependent cellular viability was examined by growing cells in galactose medium. Viable cells were observed under microscope and counted by trypan blue assay. ^∗^*p* < 0.05, significantly different when compared to indicated group. CT, control. MF, MERRF.

The cytoplasmic hybrid (cybrid) is generated by fusing cytoplast or isolated platelet with an mtDNA-depleted *ρ*^0^ cell, permitting the study of the impact of a particular mitochondrial genome on cellular physiology [27]. Our previous study has demonstrated the successful development of a stable mtDNA-depleted *ρ*^0^ cell and the creation of a cybrid cell line [24]. To establish a cybrid cell model harboring either control subjects or MERRF patients, we introduced mitochondria from control subjects and MERRF patients into mtDNA-depleted *ρ*^0^ cells (Figure 1(a)). Compared to 0% mutation of the control cybrid, the MERRF cybrid harbored more than 90% mutation of mt.8344A>G (Figures 1(b) and 1(c)). The MERRF cybrid presented increased intracellular and mitochondrial ROS expression (Figures 1(d), 1(e), and 1(f)) and a higher abundance of protein carbonylation (Figures 1(g) and 1(h)), indicating mt.8344A>G and resulting in higher cellular oxidative stress and damage. As the mitochondrial membrane potential (ΔΨm) resulting from proton gradient drives the F_o_F_1_-ATP synthase to synthesize ATP, its status regulates the balance of mitochondrial bioenergetics. To assess mitochondrial membrane potential, we used fluorescent dye TMRE that is cell permeant, and it specifically accumulates in mitochondria with membrane potential. In addition, fluorescent dye JC-1 normally presenting green fluorescent forms red fluorescent aggregates when concentrated in active mitochondria and is able to probe change of mitochondrial membrane potential. The MERRF cybrid displayed significantly abated mitochondrial membrane potential compared to the control cybrid by heat map demonstration (Figure 2(a)), as well as cytometric quantitative determination (Figures 2(b) and 2(c)). To examine respiratory capacity, cellular oxygen consumption rate (OCR) was examined using oxygen electrode chamber. Glutamate/malate, ADP, oligomycin, FCCP, and rotenone were sequentially added to cell suspension in respirometer chamber. The presence of digitonin causes the cell membrane to be permeabilized and facilitate entry of water-soluble substrates. Complex I substrate glutamate/malate is used to support basal respiration. Complex V substrate ADP is used to drive oxidative phosphorylation (OXPHOS). As ATP synthase inhibitor oligomycin specifically inhibits F_o_ portion of mitochondrial ATP synthase (complex V), the OCR suppressed by oligomycin reflects mitochondria-derived ATP turnover. Maximal respiration can be stimulated by the mitochondrial uncoupler FCCP that uncouples OXPHOS and dissipates the mitochondrial membrane potential [28]. Respiratory reserve can be accordingly determined by the difference between maximal and basal respiration. Polarography of oxygen consumption showed that the control cybrid normally consumed oxygen with an expected pattern in response to each added reagent, whereas the MERRF cybrid had a greatly diminished oxygen consumption capacity and did not respond to the presence of reagents (Figure 2(d)). Accordingly, OCR related to basal, ADP-stimulated, ATP turnover, and maximal respiration and respiratory reserve were significantly reduced in MERRF cybrids compared to that in controls (Figures 2(e), 2(f), 2(g), 2(h), and 2(i)). In addition, total ATP level of MERRF cybrids decreased approximately 80% compared to control (Figure 2(j)). The presence of oligomycin caused 80% ATP reduction in control cybrids but has no effect on MERRF cybrids (Figure 2(j)), suggesting that the MERRF cybrid loses mitochondria-derived ATP production. As MERRF mt.8344A>G affects the *MT-TK* gene that encodes mitochondrial tRNA^lys^ [13], we examined mitochondrial translation of the MERRF cybrid by analyzing the expression of mtDNA-encoded cytochrome c oxidase subunit 2 (COX2). The MERRF cybrid had a ~65% decrease in mtDNA-encoded COX2 level compared to the control cybrid, suggesting insufficient protein translation from mitochondrial genome (Figures 2(k) and 2(l)). To examine mitochondria-dependent viability, we employed glucose-free galactose medium in which cells are not able to conduct glycolysis for energy production but exclusively rely on functional mitochondria to generate energy [29]. Compared to control cybrids, MERRF cybrids showed an inability to maintain viability in a glucose-free galactose medium (Figures 2(m) and 2(n)), indicating mt8344A>G, resulting in mitochondrial dysfunction.

### 3.2. Mitochondria Transfer from WJMSC to Cells with Defective Mitochondria

**Figure 3:**
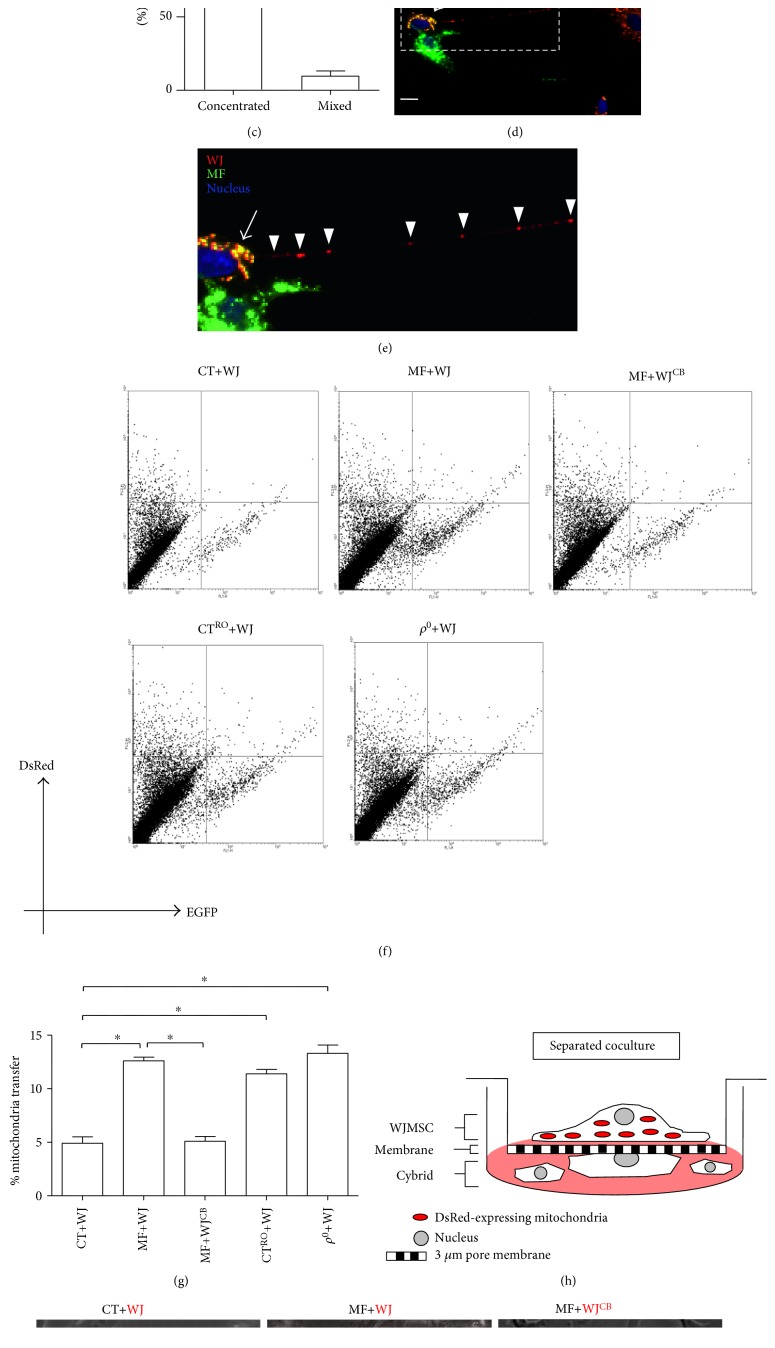
WJMSC transferred mitochondria to MERRF cybrid through a protruded tubular structure. (a) Schematic of contact coculture in which WJMSC and cybrid cell were transfected, respectively, with Cox4-DsRed and Su9-EGFP to tracking mitochondria. (b) After 24 h of coculture, images were photographed under fluorescent microscope. Scale bar, 5 *μ*m. (b′) Types of transferred mitochondria. MERRF cybrids with transferred mitochondria from WJMSC were categorized into concentrated or mixed type. Histogram data are from three experiments, with 50–100 cells counted per experiment. (c) Mitochondria of WJMSC was transferred via a protruded tubular structure to MERRF cybrid. Arrow indicates intercellular mitochondrial fusion with yellow signal. Rectangular dotted line was enlarged in (c′). (c′) Arrowheads indicate that mitochondria of WJMSC was transported along a protruded tubular structure. (d and e) Dot plot of both channels of FACS revealed percentage of mitochondrial transfer in the upper-right quadrant. (f) Schematic of separate coculture in which a 3 *μ*m pore membrane divides Cox4-DsRed-expressing WJMSC from cybrid. (g) Mitochondria from WJMSC transmitted through membrane pore was examined under fluorescence microscope after the upper chamber was offloaded. Percentage of fluorescence-positive events were counted from 25 to 40 view fields of at least three experiments. CT, control cybrid; CT^RO^, control cybrid pretreated with 500 nM rotenone for 24 h; WJ, WJMSC; MF, MERRF cybrid; MF+WJ, MERRF cybrid-plus-WJMSC; WJ^CB^, WJMSC pretreated with 350 nM cytochalasin B for 24 h.

Our previous work has revealed that WJMSC is capable of transferring healthy mitochondria to mtDNA-depleted *ρ*^0^ cells and rescues impaired mitochondrial function [20]. In light of this finding, we therefore tested whether the mitochondria of WJMSC can be transferred to the MERRF cybrid. To this end, we employed a contact coculture system in which mitochondria of both WJMSC and cybrids were individually traced by red and green fluorescent proteins (Figure 3(a)). Mitochondria-targeted fusion protein Cox4-DsRed and Su9-EGFP were, respectively, transfected into WJMSC and cybrids, followed by coculture (Figure 3(a)). After 24 h, mitochondria distribution was examined and photographed. As shown in Figure 3(b), the MERRF cybrid was noted as having received mitochondria from WJMSC (concentrated mitochondria, upper left panel, Figure 3(b)), mitochondrial mixture was shown by red-green-merged yellow signal (mixed mitochondria, upper right panel, Figure 3(b)). Although the concentrated and mixed mitochondria were simultaneously observed after 24 h coculture, the concentrated type accounted for the majority of cases of identified mitochondrial transfer (Figure 3(b′)). This phenomenon of intercellular mitochondrial transfer was not seen between WJMSC and the control cybrid (lower left panel, Figure 3(b)). Specifically, mitochondrial transfer mediated through protruded tubular structure was presented (Figures 3(c) and 3(c′)). As F-actin-composed structure can facilitate intercellular transfer of organelle, vesicles, and proteins [23, 30], we then investigated its role in WJMSC-derived mitochondrial transfer. To this end, an F-actin-depolymerizing agent cytochalasin B was used to treat WJMSC 24 h prior to coculture. As shown in Figure 3(b), mitochondrial transfer did not occur from cytochalasin B-treated WJMSC to the MERRF cybrid (lower right panel, Figure 3(b)). Furthermore, we quantitatively measured mitochondrial transfer using flow cytometry. EGFP and DsRed signal were detected using FL1 and FL2 channel. The overlapping signal in the upper right region of FL1/FL2 dot plot was defined as occurrence of intercellular mitochondrial transfer (Figure 3(d)). In like manner, WJMSC-plus-MERRF coculture had a significantly higher percentage of mitochondrial transfer than WJMSC-plus-control coculture (Figures 3(d) and 3(e)). Also, the percentage of intercellular mitochondrial transfer between WJMSC and MERRF cybrids in the presence of cytochalasin B was also abolished (Figures 3(d) and 3(e)). As the MERRF cybrids in our model demonstrated impaired mitochondrial respiration, we thus tested if suppressed mitochondrial respiration plays a role in WJMSC-derived mitochondrial transfer. To achieve this, we individually utilized rotenone and mtDNA depletion to induce mitochondrial dysfunction. Treatment of mitochondrial complex I inhibitor rotenone and mtDNA-depleted *ρ*^0^ cell were shown to cause mitochondrial dysfunction [20, 24]. As expected, the percentage of mitochondrial transfer of rotenone-treated control cybrids and *ρ*^0^ cells significantly increased compared to untreated control cybrids in coculture system with WJMSC (Figures 3(d) and 3(e)). To further confirm that WJMSC transfers mitochondria through intercellular connection but not cellular fusion, we designed a separate coculture system with a 3 *μ*m pore membrane that can permit intercellular interaction by tubular structure. As shown in Figure 3(f), WJMSC transfected with Cox4-DsRed was seeded on the upper chamber with the MERRF cybrid seeded on the bottom chamber (Figure 3(f)). The MERRF cybrid, but not the control cybrid, received mitochondria from WJMSC (Figure 3(g)). WJMSC treated with cytochalasin B was not able to transport mitochondria to the MERRF cybrid via a 3 *μ*m pore membrane (Figure 3(g)), indicating that mitochondrial transfer is dependent on F-actin-composed structure. Likewise, the control cybrid treated with rotenone and the *ρ*^0^ cell presented mitochondria transferred from WJMSC (Figure 3(g)). This result suggests that impaired mitochondrial functions, such as the status seen in mt.8344A>G, complex I inhibition, and mtDNA depletion, play a role in mitochondrial transfer from WJMSC.

### 3.3. Partly Reduced mtDNA Mutation Load by Mitochondrial Transfer Is Sufficient to Mitigate ROS Expression and Oxidative Damage and Improve Bioenergetics

**Figure 4:**
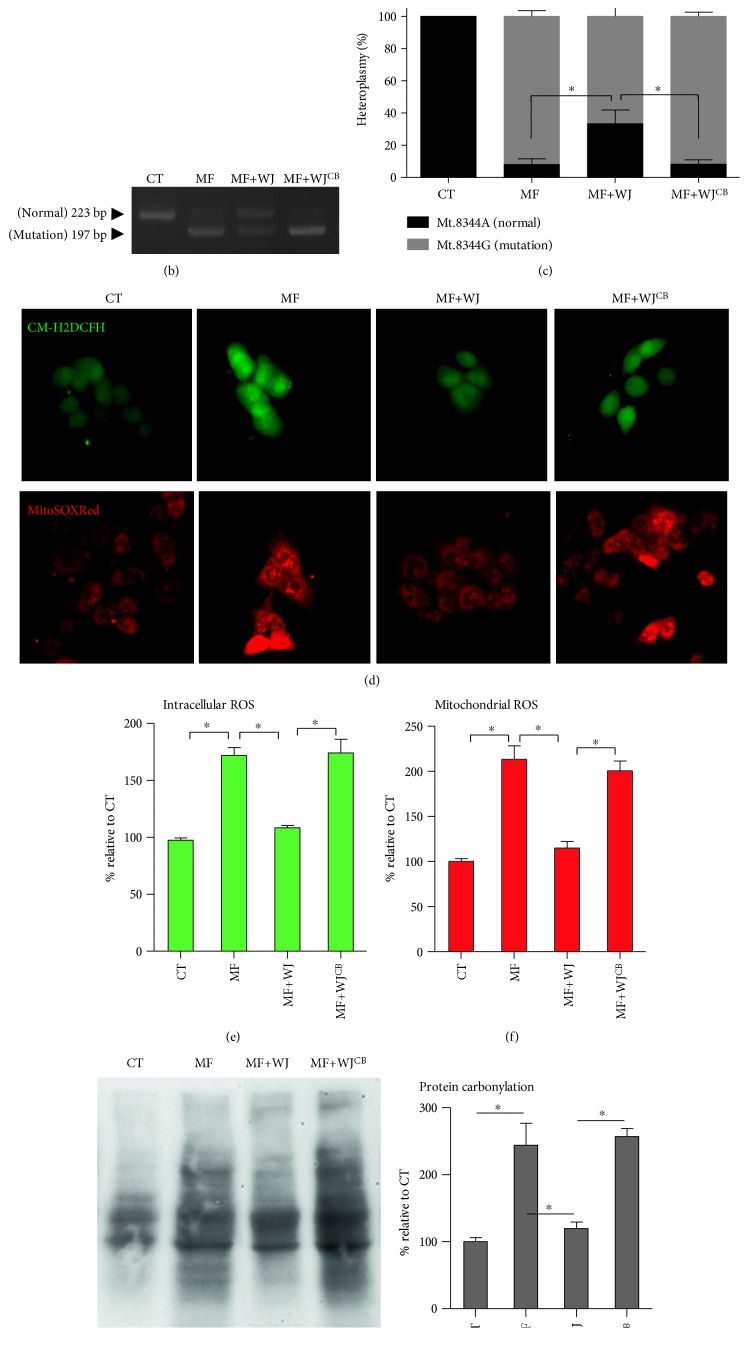
Partly reduced mtDNA mutation load by mitochondrial transfer is sufficient to mitigate ROS expression and oxidative damage. (a) Time course of coculture in which WJMSC and MERRF cybrid interacted for 7 days. Then, WJMSC was eliminated while MERRF cybrid was preserved in the presence of BrdU for another 14 days. Only cells with defective activity of thymidine kinase (TK^─^) can survive. (b-c) Mitochondrial transfer partly altered mt.8344A>G mutation rate, whereas cytochalasin B (CB) blocked the effect. (d–f) Intracellular and mitochondrial ROS expression probed, respectively, by CM-H2DCFH and MitoSOXRed were imaged with fluorescent microscope and quantified by flow cytometry. (g-h) Protein carbonylation was determined using OxyBlot method. ^∗^*p* < 0.05. CT, control cybrid; WJ, WJMSC; MF, MERRF cybrid; MF+WJ, MERRF cybrid-plus-WJMSC; WJ^CB^, WJMSC pretreated with 350 nM cytochalasin B for 24 h.

**Figure 5:**
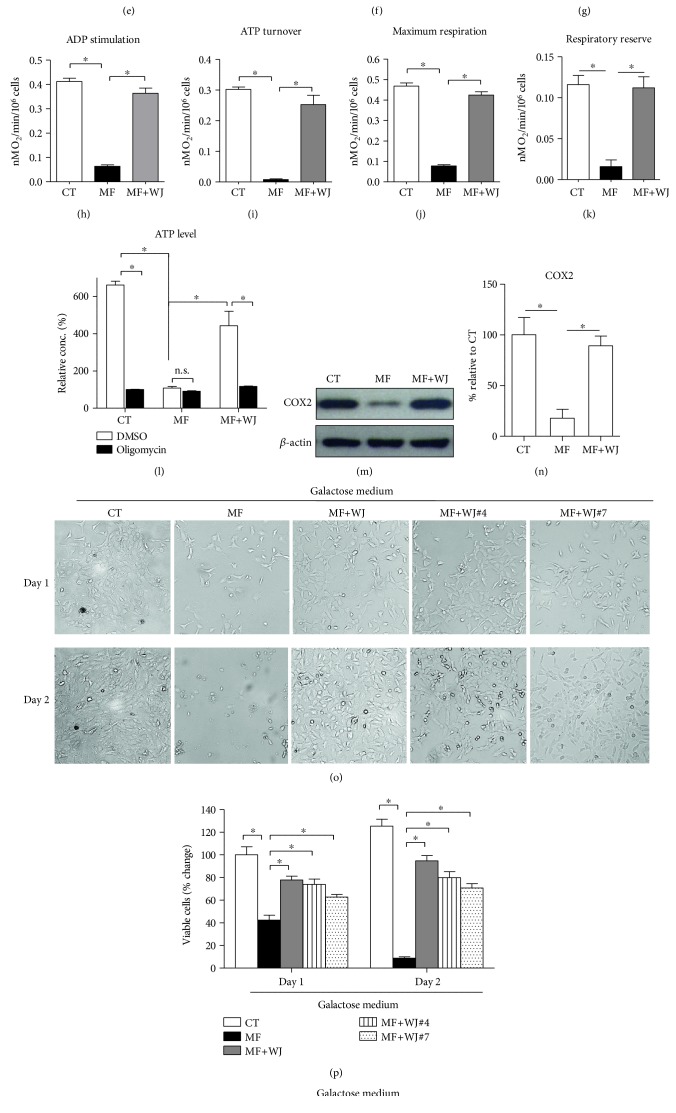
Partly reduced mtDNA mutation load by mitochondrial transfer is sufficient to improve mitochondrial bioenergetics long term. (a) Mitochondrial membrane potential probed with TMRE was imaged using a fluorescence microscope. Heat map reflecting the fluorescent intensity was analyzed using ImageJ. (b) Quantitative TMRE fluorescence was analyzed using flow cytometer. (c) Mitochondrial membrane potential probed with JC-1 was analyzed using flow cytometer. (d–f) representative polarography of OCR measurement. Substrates and inhibitors were sequentially added to a cell-containing oximeter chamber, including glutamate/malate (G/M), ADP (A), oligomycin (O), FCCP (F), and rotenone (R). (g) Basal respiration was calculated by OCR in the presence of G/M. (h) ADP-stimulated respiration was calculated by OCR in the presence of ADP. (i) Respiration related to ATP turnover was determined by the difference between ADP-stimulated OCR and oligomycin-suppressed OCR. (j) Maximal respiration was calculated by OCR in the presence of FCCP. (k) Respiratory reserve was determined by the difference between maximal and basal OCR. (l) ATP level was measured in the presence of DMSO or oligomycin. (m-n) Cytochrome c oxidase subunit 2 (COX2) expression level was determined using immunoblotting. *β*-Actin as a loading control. (o–r) Mitochondria-dependent cellular viability was examined by growing cell in galactose medium. Viable cells were observed under microscope and counted by trypan blue assay. ^∗^*p* < 0.05, significantly different when compared to indicated group. CT, control; MF, MERRF; MF+WJ, MERRF cybrid-plus-WJMSC.

To inspect mitochondrial function and cellular performance of the MERRF cybrid following mitochondrial transfer from WJMSC, we employed BrdU to remove WJMSC and preserve the MERRF cybrids in coculture system, as WJMSC could not survive with the presence of BrdU. As described in our previous work, cybrids with nuclear background of 143B osteosarcoma have defective activity of thymidine kinase (TK^─^) and hence present resistance against BrdU [20]. Then, we cocultured the MERRF cybrid (MF) with WJMSC (WJ) for seven days, followed by 14 days of BrdU selection to remove WJMSC. The remaining cells (MF+WJ cells) should contain normal functional mitochondria and 143B nucleus background which further demonstrates that after coculture MERRF cybrids received mitochondrial transfer from WJMSC (Figure 4(a)). MF+WJ cells demonstrated a partly reduced mtDNA mutation load, whereas WJMSC pretreated with cytochalasin B did not show mitochondrial transfer to affect mtDNA mutation load (Figures 4(b) and 4(c)). The expression of intracellular and mitochondrial ROS (Figures 4(d), 4(e), and 4(f)) and abundance of protein oxidation (Figures 4(g) and 4(h)) were suppressed by the reduction of mtDNA mutation load. Furthermore, mitochondrial membrane potential of MF+WJ cells was partly recovered (Figures 5(a), 5(b), and 5(c)). OCR of basal status, ADP-stimulated, ATP turnover, maximal activity, and respiration reserve of MF+WJ cells were recaptured (Figures 5(d), 5(e), 5(f), 5(g), 5(h), 5(i), 5(j), and 5(k)). ATP level of MF+WJ cells was significantly recovered and mitochondrial ATPase inhibitor oligomycin caused ATP level to plummet (Figure 5(l)). MtDNA-encoded COX2 expression of MF+WJ cells was reversed (Figures 5(m) and 5(n)). In contrast to the inability of the MERRF cell to survive the galactose medium, the MF+WJ cell was able to expand in a glucose-free condition (Figures 5(o) and 5(p)). Moreover, we demonstrated that three lines of WJMSC from different donors were able to rescue mitochondria-dependent viability of MERRF cells (Figures 5(o) and 5(p)). Notably, the recaptured mitochondria-dependent viability persisted for a further 60 days of cultivation, more than 20 passages (Figures 5(q) and 5(r)). These results indicated that a partly reduced mtDNA mutation load by mitochondrial transfer is sufficient to mitigate ROS expression and oxidative damage and improve bioenergetics.

### 3.4. Improvement of Mitochondrial Bioenergetics and Oxidative Stress Contributes to Recapture of Mitochondrial Network and Apoptotic Tolerance

**Figure 6:**
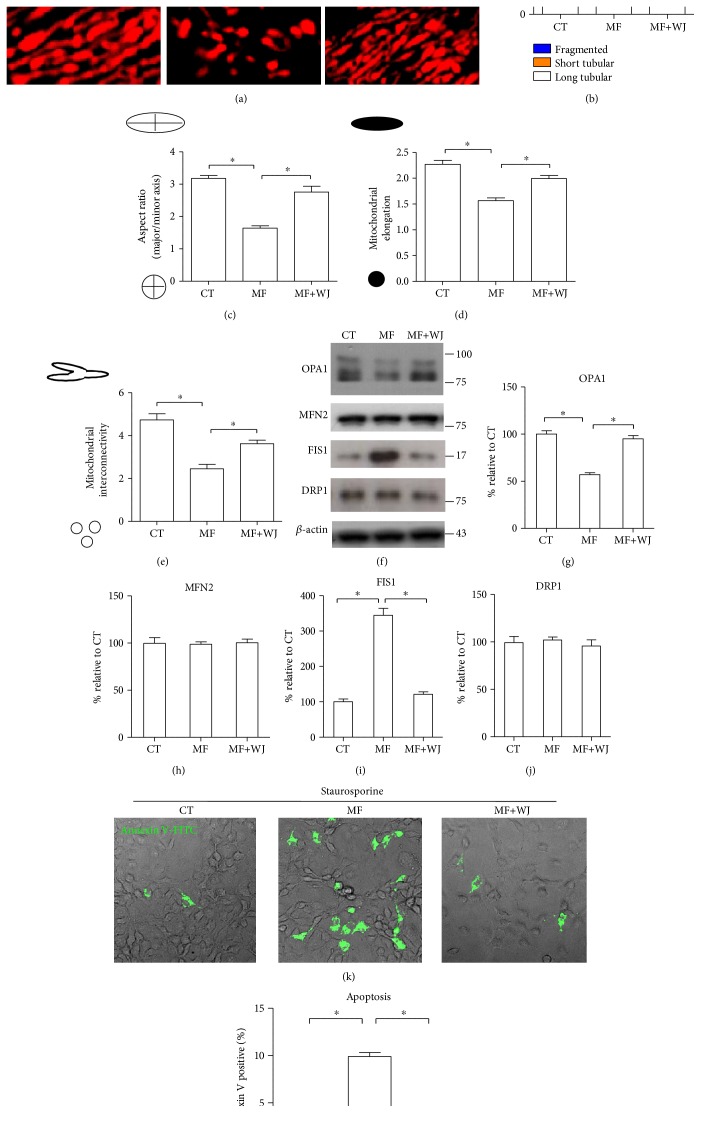
Improvement of mitochondrial bioenergetics and oxidative stress is associated with recapture of mitochondrial network and antiapoptosis resistance. (a) Cells expressed mitochondrial Cox4-DsRed to display mitochondrial morphology under confocal microscope. Scale bar, 5 *μ*m. (b) 50–100 cells were counted for each cell type. (c–e) Mitochondrial morphology was analyzed using Dagda's method [26], as described in Materials and Methods. (f–j) Expression level of mitochondrial fusion protein (OPA1 and MFN2) and fission protein (FIS1 and DRP1). (k-l) Cellular apoptosis was induced by 6 h exposure of 500 nM staurosporine. Apoptosis was detected by Annexin V-FITC. ^∗^*p* < 0.05, significantly different when compared to indicated group. CT, control; MF, MERRF; MF+WJ, MERRF cybrid-plus-WJMSC.

To analyze mitochondrial network and dynamics, cells were transfected with mitochondrial Cox4-DsRed to display mitochondria. Compared to that of the control cybrid, the MERRF cybrid predominantly presented fragmentation of mitochondria (Figures 6(a), 6(b), 6(c), 6(d), and 6(e)). In line with the fragmented shape of mitochondria, the MERRF cybrid presented decreased OPA1 and increased FIS1 but no change in MFN2 and DRP1 (Figures 6(f), 6(g), 6(h), 6(i), and 6(j)). Also, the MERRF cybrid was more susceptible to apoptosis inducer staurosporine (STS) than the control cybrid (Figures 6(k) and 6(l)). Moreover, mitochondrial network, fusion/fission protein expression pattern, and STS susceptibility were recaptured in MF+WJ cells (Figure 6), suggesting that improvement of mitochondrial bioenergetics and oxidative stress by mitochondrial transfer contributes to modifications in mitochondrial dynamics and apoptotic tolerance.

## 4. Discussion

Although it has been established that mtDNA mutation causes MERRF, the MERRF cybrid model was constructed to confirm the causal relationship between mutation and mitochondrial dysfunction; a feasible strategy to rescue disease progression remains wanting. Here, we visualized and quantified that WJMSC is able to conduct mitochondrial transfer along with wild-type mtDNA into the mt.8344A>G harboring the MERRF cybrid, resulting in a partial reduction of mtDNA mutation load, oxidative stress, and mitochondrial bioenergetics. Following improved mitochondrial function, the MERRF cybrid presents amelioration in mitochondria-dependent cellular viability, mitochondrial networks and dynamics, and antiapoptosis resistance. These results indicate a possible therapeutic strategy for managing MERRF syndrome.

There is some evidence revealing that exogenous donation of normal mitochondria is able to improve mitochondrial disorders that are caused by either mtDNA mutation or stress. Peptide-mediated mitochondrial delivery has been shown to be effectively transferred into human cells harboring mt.8344A>G in vitro and to improve mitochondrial protein synthesis, bioenergetics, biogenesis, dynamics and calcium homeostasis, while reducing ROS generation [31, 32]. Intercellular mitochondrial transfer via tunneling nanotubes is able to rescue UV-damaged rat pheochromocytoma cells from apoptosis in vitro. In addition, mitochondrial transfer from BMMSC to LPS-damaged lung alveolar epithelia in vivo significantly reduces acute lung injury and increases alveolar bioenergetics, as well as improves survival rate [21]. Recently, we also found that human WJMSC demonstrates the capability to transfer mitochondria to mtDNA-devoid cells and recapture mitochondrial protein synthesis and bioenergetics. In this study, we observed the transfer of mitochondria from WJMSC to MERRF cybrids which mitigated mtDNA mutation load, oxidative stress, and mitochondrial bioenergetics. Thus, our study provides further support for the notion that successful delivery of normal mitochondria along with mtDNA can rescue mitochondrial dysfunction.

MtDNA heteroplasmy is a critical contributor to the severity of mitochondrial diseases. Our results demonstrate that mitochondrial transfer was coupled with mtDNA transfer, which partly reduced mt.8344A>G mutation load. This reduced mtDNA mutation burden may contribute to the enhancement of translation of mtDNA-encoded protein. We reason that corrected mitochondrial translation assists respiratory enzyme complex to perform mitochondrial bioenergetics, such as facilitating electron transport, generating mitochondrial membrane potential, oxygen consumption, and ATP production through oxidative phosphorylation. Accordingly, the clinical application of WJMSC for MERRF and other mitochondrial disease patients may offer potential for the development of therapeutic treatment. Besides cells harboring mt.8344A>G, we also demonstrated that WJMSC transfers mitochondria to cells exposed to mitochondrial complex I inhibitor rotenone, as well as those with depleted mtDNA. That may raise the prospect that WJMSC possesses therapeutic potential for other diseases associated with mitochondrial disorders, such as Parkinson disease, Huntington disease, and metabolic diseases.

Whether the effect of mitochondrial transfer can persist for the long term may be a critical issue for the development of future clinical applications. Chang and colleagues showed that the effect of peptide-mediated mitochondria delivery to MERRF cells can be maintained for at least 21 days [32]. Here, we demonstrated that mitochondrial transfer from WJMSC to the MERRF cell sustains for at least 60 days, and aerobic viability of rescued MERRF cells still remains intact. These findings imply that the MERRF cell is likely to preserve transferred mtDNA without immediately removing exogenous mtDNA. Interestingly, Cho et al. demonstrated that bone marrow mesenchymal stem cells can transfer mitochondria to *ρ*^0^ cells but not to cybrid cells harboring the pathogenic mutation of mt.3243A>G and 4977 bp deletion. Although the issue of whether bone marrow mesenchymal stem cells can transfer mitochondria to MERRF cells is not investigated in Cho's study, the mechanism receiving exogenous mitochondria may be different between mt.8344A>G and mt.3243A>G/4977 bp deletion. On the other hand, the possibility that WJMSC may possess a superior ability to transfer mitochondria cannot be ignored. In this regard, Lund et al. have shown that WJMSCs exhibited superior efficacy than bone marrow mesenchymal stem cells in rescuing the photoreceptor after transplantation into the rodent's eyes [33]. More studies are required to determine the efficiency and rescue effect of WJMSC compared to those of other cell sources.

Mitochondrial matrix-localized fluorescent protein facilitates the surveillance of mitochondrial transfer, as well as fusion status of mitochondria from two cellular origins. We employed matrix-localized Cox4-DsRed and Su9-EGFP to demonstrate not only intercellular mitochondrial transfer but also the integrating status of both mitochondria (Figure 3). We observed two distinct patterns of transferred mitochondria, concentrated and mixed mitochondria, which take place simultaneously, with the concentrated pattern as the majority (Figures 3(b) and 3(b′)). In this regard, concentrated and mixed presentations indicate incomplete and full fusion of both mitochondria, respectively. We reason that transferred mitochondria from WJMSC in an earlier phase are yet to fuse with MERRF cybrid's mitochondria, demonstrating a concentrated appearance; whereas in a later phase, both mitochondria undergo full fusion, resulting in a mixed appearance. As a result, the reciprocal fusion may allow modification of mtDNA heteroplasmy. Accordingly, the reduction of mtDNA mutation load in MERRF cybrid receiving mitochondrial transfer verifies the sustainability of WJMSC mtDNA (Figures 4(b) and 4(c)).

WJMSC exhibits similar basic hallmarks of adult MSCs, such as surface markers, morphology, and multipotency to differentiation [34]. Adult MSCs, such as bone marrow MSC and adipocyte-derived MSC, have been shown to conduct mitochondrial transfer to restore cellular viability [21, 35, 36]. However, the procedure to obtain adult MSC is highly invasive and not frequently acceptable for healthy donors. Unlike adult MSC, WJMSC is derived from the umbilical cord, which is a natural form of waste resulting after birth and free from additional invasive procedures. This makes WJMSC a more accessible source of stem cells for use in clinical and experimental applications. Harvesting WJMSC allows for a rapid initial isolation of larger cell numbers and growth of WJMSC demonstrates shorter doubling time compared to BMMSCs [37, 38]. Of note, the immunomodulatory properties of WJMSC that avoids graft-versus-host disease and permits transplanted cells to survive long term [39] allow for possible immunosuppressant-free management following xenograft transplantation. Additionally, the inability of WJMSC to perform attachment-free colony formation and invasiveness in vitro implies that it is devoid of tumorigenic potential [20]. These advantages thus facilitate the further development of WJMSC-based therapy and may pave the way for future clinical application.

With reference to the mechanisms accounting for mitochondrial transfer, previous studies have suggested that the induction of mitochondrial dysfunction and the formation of F-actin-containing tunneling nanotubes may play a role. In this regard, Ahmad et al. demonstrated that mitochondrial dysfunction in epithelial cell injury induced by mitochondrial complex I inhibitor rotenone can be rescued by BMMSC-derived mitochondrial transfer via intercellular F-actin-containing tubular structure [22]. Knockdown tunneling nanotube-formatting protein TNFAIP2 abolished mitochondrial transfer and lead to a substantial reduction of therapeutic effect [22]. On the other hand, Wang and Gerdes have shown that UV light-stressed pheochromocytoma cells form tunneling nanotubes that communicate with healthy cells and facilitate mitochondrial transfer to prevent UV stress-induced apoptosis [23]. F-actin-depolymerizing agent cytochalasin B abolished intercellular formation of tunneling nanotubes and accordingly blocked mitochondrial transfer and antiapoptosis effect [23]. In our study, mitochondrial transfer from WJMSC is able to rescue mitochondrial dysfunction caused by the mt.8344A>G mutation in MERRF cells. We demonstrated the occurrence of mitochondrial transfer by observing and quantifying fluorescence mitochondria in a contact coculture system, as well as a separated coculture system which permits tubular structure-mediated communication without cell-cell contact. In addition, mitochondrial transfer from WJMSC occurred when cocultured with cells stressed with rotenone and those devoid of mtDNA but not cells with functional mitochondria. Moreover, cytochalasin B pretreatment to WJMSC abolished the mitochondrial transfer and subsequent rescue effect. These results support the notion that induction of mitochondrial dysfunction and the formation of F-actin-containing tunneling nanotubes play a role in the process of mitochondrial transfer.

In addition to bioenergetics, the mt.8344A>G mutation in MERRF cells also leads to higher oxidative stress. MERRF cybrids and fibroblasts were reported to present increased intracellular H_2_O_2_ and oxidation-related markers [15]. Higher oxidative stress may render the MERRF cell more sensitive to the apoptotic inducer staurosporine, as shown in our study. In this regard, perturbed calcium homeostasis induced by mt.8344A>G-associated mitochondrial dysfunction and subsequent altered activity of calpain protease play a role in the hypersensitivity of MERRF cells to staurosporine [40]. As well, induced oxidative stress caused mitochondrial fragmentation [41], whereas OPA1 overexpression reduces intracellular oxidative stress and cellular apoptosis [42], suggesting the existence of mutual regulation between mitochondrial dynamics and oxidative stress. Our study reveals that the MERRF cybrid receiving WJMSC-derived mitochondrial transfer exhibited a reduced ROS level, increased mitochondrial fusion-network and profusion manner of dynamic protein, and enhanced resistance against apoptotic stress. We reason that improved mitochondrial bioenergetics through the reduction of heteroplasmic mtDNA mutation load following mitochondrial transfer plays a role in remodeling the elongated morphology of mitochondria. Indeed, OPA1-mediated mitochondrial fusion required functional OXPHOS [43]. We show that the MERRF cybrid with impaired OXPHOS presents reduced OPA1 and increased FIS1 expression along with fragmented mitochondria. After mitochondrial transfer, the corrected OXPHOS activity of the MERRF cybrid may contribute to a recovered OPA1/FIS expression pattern, which is then responsible for elongated morphology of mitochondria. Consistent with our data, Chang et al. have demonstrated that fragmentation of mitochondria network and decreased OPA1/increased FIS1 expression were caused by the mt.8344A>G mutation in MERRF cybrids, while mitochondrial transfer conducted by peptide-mediated mitochondrial delivery restored the network morphology and OPA1/FIS expression [31].

**Figure 7:**
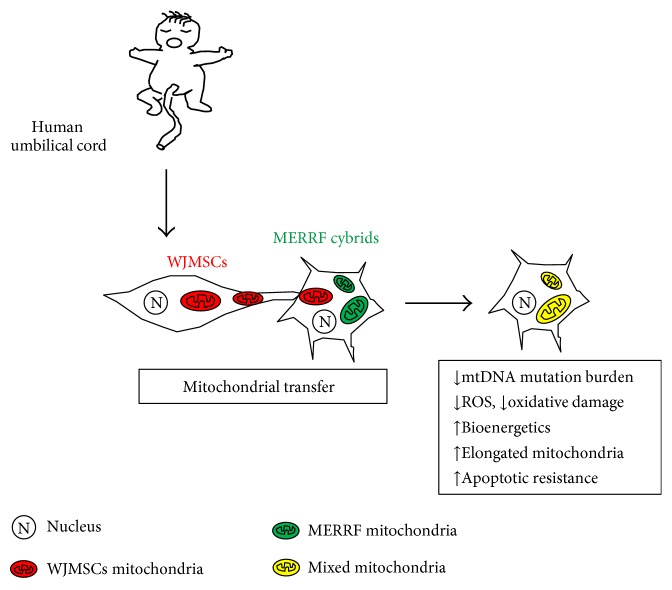
Graphic summary: WJMSC is capable of transferring healthy mitochondria to MERRF cybrid and reduces mtDNA mutation burden. Improved mtDNA mutation burden leads to amelioration in ROS generation, oxidative damage, and mitochondrial bioenergetics. The therapeutic effect of mitochondrial transfer also contributes to elongated network of mitochondria and apoptotic resistance.

This study provides evidence for successful mitochondrial transfer from WJMSC to MERRF cybrids and its subsequent rescue effect in the changes to mtDNA mutation load, oxidative stress, bioenergetics, mitochondrial dynamics, and antiapoptotic resistance (Figure 7). We propose a new stem cell-based mitochondrial transfer resource for rescuing cells with mitochondrial disorders, which offers promise for the further development of treatment for mitochondrial diseases.
